# The Inhibition of the Dorsal Paragigantocellular Reticular Nucleus Induces Waking and the Activation of All Adrenergic and Noradrenergic Neurons: A Combined Pharmacological and Functional Neuroanatomical Study

**DOI:** 10.1371/journal.pone.0096851

**Published:** 2014-05-08

**Authors:** Olivier Clément, Sara Valencia Garcia, Paul-Antoine Libourel, Sébastien Arthaud, Patrice Fort, Pierre-Hervé Luppi

**Affiliations:** 1 INSERM, U1028; CNRS, UMR5292; Lyon Neuroscience Research Center, Team SLEEP, Lyon, France; 2 University Claude Bernard Lyon 1, Lyon, France; The Research Center of Neurobiology-Neurophysiology of Marseille, France

## Abstract

GABAergic neurons specifically active during paradoxical sleep (PS) localized in the dorsal paragigantocellular reticular nucleus (DPGi) are known to be responsible for the cessation of activity of the noradrenergic neurons of the locus coeruleus during PS. In the present study, we therefore sought to determine the role of the DPGi in PS onset and maintenance and in the inhibition of the LC noradrenergic neurons during this state. The effect of the inactivation of DPGi neurons on the sleep-waking cycle was examined in rats by microinjection of muscimol, a GABA_A_ agonist, or clonidine, an alpha-2 adrenergic receptor agonist. Combining immunostaining of the different populations of wake-inducing neurons with that of c-FOS, we then determined whether muscimol inhibition of the DPGi specifically induces the activation of the noradrenergic neurons of the LC. Slow wave sleep and PS were abolished during 3 and 5 h after muscimol injection in the DPGi, respectively. The application of clonidine in the DPGi specifically induced a significant decrease in PS quantities and delayed PS appearance compared to NaCl. We further surprisingly found out that more than 75% of the noradrenergic and adrenergic neurons of all adrenergic and noradrenergic cell groups are activated after muscimol treatment in contrast to the other wake active systems significantly less activated. These results suggest that, in addition to its already know inhibition of LC noradrenergic neurons during PS, the DPGi might inhibit the activity of noradrenergic and adrenergic neurons from all groups during PS, but also to a minor extent during SWS and waking.

## Introduction

Since the discovery of paradoxical (PS) sleep (also known as REM sleep) more than fifty years ago, a large number of studies have been devoted to the identification of the neuronal network responsible for its genesis. In the recent years, it has been reported that many structures located from the hypothalamus to the medulla oblongata contain neurons involved in PS regulation [1]–[3]. Among these structures, the dorsal paragigantocellular reticular nucleus (DPGi), localized in the dorsomedial part of the medulla oblongata, might play a key role in PS control.

It has indeed been shown that, in rats, the DPGi contains a large population of neurons expressing c-FOS (a marker of neuronal activation) during PS hypersomnia [4]. In addition, electrophysiological recordings in head-restrained rats confirmed the presence in the DPGi of neurons strongly active specifically during PS (PS-on neurons) [5]. It has then been shown that 80% of the DPGi neurons expressing c-FOS during PS hypersomnia also express glutamate decarboxylase 67, the enzyme of synthesis of GABA [6]. A number of studies strongly suggest that these GABAergic PS-on neurons are responsible for the cessation of activity during PS of the wake-active neurons of the locus coeruleus (LC). Combining unit recording of LC noradrenergic neurons and bicuculline iontophoresis, it has indeed been demonstrated, in agreement with a microdialysis study [7], that LC noradrenergic neurons are tonically inhibited by GABA during PS [8]. Further, in anaesthetized rats, electrical stimulation of the DPGi region inhibits with a short latency the wake-active LC noradrenergic neurons by means of GABAergic mechanisms [9]. In addition, electrical stimulation of the DPGi in freely moving rats induces a two fold increase in PS quantities that is reversed by simultaneous application in the LC of picrotoxin (a GABA_A_ antagonist) [10]. It has then been shown in rats that the DPGi contains the majority of neurons projecting to the LC and expressing c-FOS (a marker of neuronal activation) during PS hypersomnia [11]. All these data strongly suggest that PS-on GABAergic neurons located in the DPGi are responsible for the cessation of activity of LC noradrenergic neurons during PS.

In addition to the LC, the DPGi projects to other areas containing wake-promoting neurons such as the serotoninergic neurons of the dorsal raphe nucleus, the pontine cholinergic neurons of the laterodorsal tegmental nucleus and, to a small extent, the hypocretin neurons of the posterior hypothalamus [5], [12], [13]. These results suggest that DPGi GABAergic PS-on neurons could be responsible for the inactivation, during PS, of the LC noradrenergic cells and also for that of other types of wake-active neurons.

First, to determine whether inactivation of cells in the DPGi region suppresses the occurrence of PS, we injected two inhibitory compounds, muscimol (a GABA_A_ receptor agonist) and clonidine hydrochloride (an alpha-2 adrenergic receptor agonist) in the DPGi in freely moving rats and recorded the sleep-wake cycle during 8 h. Muscimol was chosen because all neurons are known to express GABA_A_ receptors. Clonidine was also used because noradrenergic neurons are known to be selectively active during waking and could provide an inhibitory feedback to the GABAergic PS-on neurons of the DPGi. Further, the expression of alpha2 adrenergic receptors has been reported in the region of the DPGi [14], [15]. Second, to determine whether the loss of sleep caused by inactivation of cells in the DPGi region is associated with activation of some, or all, wake-active neuronal groups, we combined the use of c-FOS as a marker of neuronal activation with the immunostaining of markers of the wake-active systems (noradrenergic, adrenergic, dopaminergic, serotoninergic, cholinergic, hypocretinergic and histaminergic).

## Methods

### Ethics Statements

All experiments were conducted in accordance to the European Community Council Directive for the use of research animals (86/609/EEC) and approved by the institutional animal care and use committee of the University of Lyon 1 (Comité d'expérimentation animale de l'Université Claude Bernard Lyon 1). Sprague-Dawley male rats were housed individually in recording barrels under a constant 12 h light-dark cycle (light on at 08∶00 AM). Room temperature was maintained at 21±1°C, and standard rodent food and water were available *ad libitum* throughout the experiment.

### Cannula and EEG/EMG Implantation

Rats (n = 13, 290–300 g, Charles River) were implanted for cannula injection inside the DPGi. The cannula targeted the midline of the DPGi at the following stereotaxic coordinates (anterior (A)-posterior (P), −12; dorsal (D)-ventral (V), −7.8; distances in mm from bregma, except for DV which is from the brain surface). To this aim, rats were anesthetized with a Ketamine/Xylazine mix (100 mg.kg^−1^ and 10 mg.kg^−1^ respectively, i.p.) and mounted in a stereotaxic frame (David Kopf Instruments). A 26 gauge guide cannula (Plastics One Inc.) was first implanted 2.6 mm above the DPGi and secured to the skull using acrylic (Superbond, Sun Medical Co. Ldt.) and dental (Paladur, Heraeus Kulzer) cement. Then, all rats were implanted for electroencephalographic (EEG) and electromyographic (EMG) recordings. Briefly, for EEG, three stainless-steel screws (Anthogyr) were inserted over the frontal (AP, +3; ML, +1), occipital (AP, −8; ML, +3) and cerebellar (AP, −12; ML, +2) cortices. Two gold-coated electrodes were also inserted into the neck muscles in order to record EMG activity. All EMG and EEG electrodes were then fixed to the skull using acrylic cement (Superbond) and connected to a miniature plug (Plastics One Inc.) that was cemented using dental cement (Paladur).

### Pharmacology

Animals were allowed to recover from surgery for 5 days during which they were weighed daily. They were then habituated to the recording conditions and handled daily for at least 15 min during one week to minimize non-specific stress. 0.3 µl of muscimol (a GABA_A_ receptor agonist, 1 µg.µl^−1^ in NaCl; Sigma-Aldrich), clonidine hydrochloride (an alpha-2 adrenergic receptor agonist, 0.53 µg.µl^−1^ in NaCl; Tocris Bioscience) or NaCl (0.9%) were injected at the rate of 0.1 µl.min^−1^ using an Hamilton syringe (Hamilton) connected to the cannula with the appropriate polyethylene tubing (Plastics One Inc.) and inserted into a micro-pump (New Era Pump System). The 33 gauge cannula (Plastics One Inc.) protruded out 2.6 mm from the guide cannula.

All injections started at 9∶00 AM. Cannula was lowered down the brain and left in place for 5 min before and after the injection. Animals were then put back in their barrels and vigilance states were recorded for 8 h. All drugs were stored at final concentration at −20°C and one new batch was used for each injection. In addition to a control injection of NaCl, each rat received a muscimol and/or a clonidine injection. Two consecutive injections were spaced by at least 3 days and the sequence of drug injections was randomized. Finally, for the c-FOS study (see below), 8 rats received a last injection of either muscimol (n = 4) or NaCl (n = 4) and were sacrificed 3 h after the end of the injection.

### Polygraphic Recordings

Rats were connected to a cable attached to a rotating connector (Plastics One Inc.) to allow free movements of the animal within its cage. EEG and EMG signals were amplified (MCP+, Alpha-Omega Engineering) and collected on a computer via a CED interface using Spike2 software (Cambridge Electronic Design). For all animals, the same signal amplification was used and each animal remained in the same recording box during the entire experiment. Sampling frequency for polygraphic recordings was 520.8 Hz.

### Analysis of the Sleep-wake Data

#### Quantification of the sleep-wake cycle

The sleep-wake cycle was analysed for 8 h following injection. Blind to experimental conditions, vigilance states were manually scored by 5 s epochs using a homemade script developed for Spike2 software (Cambridge Electronic Design). State classification was done by visual examination of polygraphic signals and the help of a sliding window showing the EEG power spectrum analyzed by Fast Fourier Transform during the same 5 s epoch. Wake (WK) was characterized by desynchronized (activated) low-amplitude EEG accompanied by a sustained EMG activity; SWS was distinguished by high-voltage slow waves (1.5 – 4.0 Hz), spindles (10 – 14 Hz) and the disappearance of phasic muscle activity. The occurrence of muscle atonia with a regular and pronounced theta rhythm (4 – 9 Hz) characterized PS. We then calculated the quantities, percentages, numbers and duration of bouts for each vigilance state. SWS and PS latencies, defined as the time between the end of the injection session and the first SWS or PS episode, respectively, were also calculated.

#### WK spectral analysis

Spectral analysis for the characterization of WK was done on occipital EEG signals following muscimol and NaCl injections. All artefacted epochs were excluded from the analysis. The spectral analysis of WK was done during 8 h by 20 min periods. For each 5 s epoch of WK, a power spectrum was calculated by Fast Fourier Transform (bin size 0.25 Hz, from 0.6 Hz to 45 Hz). Then, for each animal and for each 20 min period, a mean spectrum over all WK epochs was calculated, and values after muscimol injection were normalized to NaCl ones.

### Perfusion, Fixation and Tissue Sectioning

8 rats were sacrificed 3 h after injection of either muscimol or NaCl in the DPGi. Under deep pentobarbital anaesthesia (Ceva santé animale, 150 mg.kg^−1^, i.p.), rats were transcardially perfused with Ringer’s lactate solution containing 0.1% heparin, followed by 500 ml of a cold fixative solution composed of 4% paraformaldehyde in 0.1 M phosphate buffer (PB, pH 7.4). Brains were removed and stored at 4°C overnight in the fixative solution and then for 3 days in 30% sucrose in 0.1 M PB. Brains were then rapidly frozen in methyl-butane cooled with dry ice and 30 µm thick coronal sections were cut on a cryostat (Microm).

### Immunohistochemistry

As previously described [4], [16], brain sections were successively incubated in (i) a rabbit anti-serum against c-FOS (1∶10,000; Merck, catalogue number PC38) in PBST-Az (PBST containing 0.1% of azide) for 3 days at 4°C; (ii) a biotinylated goat anti-rabbit IgG solution (1∶1,000; Vector Laboratories); and (iii) an ABC-HRP solution (1∶1,000; Elite kit, Vector Laboratories) both for 90 min at room temperature. Finally, the sections were immersed in a 0.05 M Tris-HCl buffer (pH 7.6) containing 0.025% 3,3'-diaminobenzidine-4 HCl (DAB; Sigma-Aldrich), 0.003% H_2_O_2_ and 0.6% nickel ammonium sulphate. Three PBST washes of 10 min were performed between each step. The c-FOS stained sections were then incubated the following day in either: a goat polyclonal antibody against hypocretin (HCRT) (1∶5,000; Santa Cruz Biotechnology Inc., catalogue number sc-8070), a goat polyclonal antibody against choline acetyltransferase (ChAT) (1∶2,000; Millipore, catalogue number AB144P), a mouse monoclonal antibody against serotonin (5-HT) (1/300,000, generated by Y. Charnay, [17], a rabbit antibody against tyrosine hydroxylase (TH) (1∶5,000; Institut de Biotechnologies Jacques Boy, catalogue number 208020234), or a rabbit antibody against histidine decarboxylase (HDC) (1∶10,000; Acris Antibodies, catalogue number EUD2601) in PBST-Az over 3 days at 4°C. Amplification steps were similar to those described above except that the revelation was performed in the absence of nickel. Finally, the sections were mounted on glass slides, dried and coverslipped with DePeX.

### Quantitative Analysis of Labeled Neurons Distribution

For each neuronal population analyzed (TH, 5-HT, ChAT, HCRT and HDC), we counted on hemi-sections the singly labeled neurons (TH+ for example) and those expressing c-FOS (TH/c-FOS+ for example) in all structures containing the given type of neuron (adrenergic and noradrenergic cell groups for example). To cover the entire rostro-caudal extent of each structure, sections were counted every 600 µm for TH, 360 µm for 5-HT, ChAT and HCRT and 180 µm for HDC labelling. The distribution of c-FOS labelled cells was also analysed on nine sections taken every 360 µm at the level of the DPGi. Drawings of labelled cells were made with an Axioscope microscope (Zeiss) equipped with a motor ized X–Y-sensitive stage and a video camera connected to a computerized image analysis system (Mercator, ExploraNova). The number of single and double-labeled neurons per structure was automatically counted and exported using Mercator (ExploraNova). All the analysis were done blind to the experimental conditions.

### Statistical Analysis

Because of the small number of animals used in each experiment (n = 8 for each group in the pharmacological study and n = 4 for each group in the c-FOS study), it was not possible to test for the normality of the data. As a consequence, we used non-parametric tests. The number of labeled neurons across experimental conditions was compared using a Mann-Whitney *U* test. The effects of pharmacological treatments on the sleep-wake cycle (quantities, latencies, power spectra etc.) were analysed with a Wilcoxon signed rank test. All statistics were performed using StatView software and a significant effect was considered for all p<0.05.

## Results

### Muscimol Injection into the DPGi Induces Wakefulness and Delays PS

**Figure 1:**
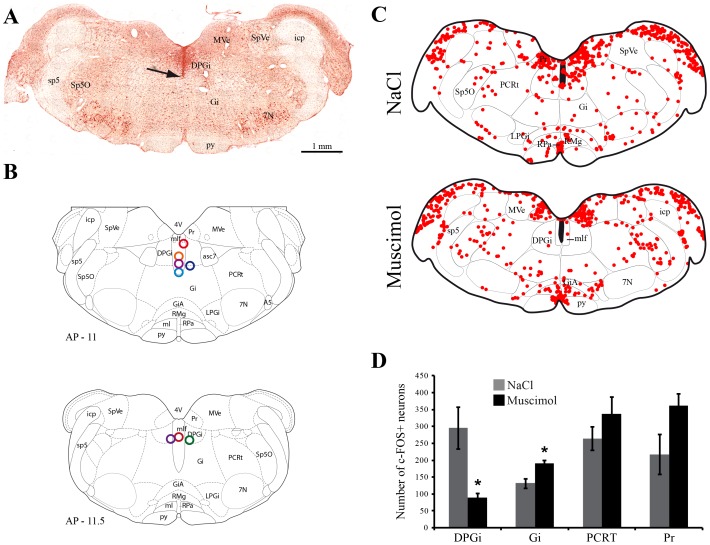
Muscimol injection into the DPGi reduces c-FOS expression specifically within the DPGi. For each animal, the injection site was localized at the end of the protocol. **A**, Example of a DPGi injection site on a neutral red counterstained section. The tip of the cannula is indicated by a black arrow. **B**, Drawing showing the localization of all injection sites. Adapted from Paxinos and Watson Rat Brain atlas, 5^th^ edition. The antero-posterior localization of each drawing is indicated at the left bottom corner in mm from the bregma. **C**, Drawings showing the distribution of c-FOS positive neurons (red dots) on a section at the level of the injection site in two representative animals perfused 3 h after NaCl ormuscimol injection in the DPGi. The black filled area represents the cannula trace. **D**, Number of c-FOS+ neurons counted in the DPGi and in the surrounding regions after muscimol and NaCl injection in the DPGi. Values are mean ± SEM, n = 4 in each group, *p<0.05 between muscimol and NaCl. Abbreviations: 7N, facial nerve; 4V, 4th ventricle; DPGi, dorsal paragigantocellular reticular nucleus; Gi, gigantocellular reticular nucleus; GiA, gigantocellular reticular nucleus, alpha part; icp, inferior cerebellar peduncle; LPGi, lateral paragigantocellular reticular nucleus; mlf, medial longitudianl fasciculus; MVe, medial vestibular nucleus; PCRt, parvicellular reticular nucleus; Pr, prepositus nucleus; py, pyramidal tract; RMg, raphe magnus nucleus; RPa, raphe pallidus nucleus; sp5, spinal trigeminal tract; Sp5O, spinal trigeminal nucleus, oral part; SpVe, spinal vestibular nucleus.

In all animals (n = 8), the injection sites were located in the middle part of the DPGi (Figure 1*A,B*). In animals perfused 3 h after the last muscimol injection, the analysis of c-FOS labelling in and around the DPGi revealed that the number of c-FOS+ neurons was significantly and specifically reduced in the DPGi when compared to NaCl (p = 0.021), whereas it was significantly increased in the Gi (p = 0,043) and tended to increase in the Pr and PCRt (p = 0.15 and p = 0.39 respectively) (*Figure 1C*, D). In addition, the prepositus hypoglossi, the raphe pallidus and magnus nuclei as well as the GiA contain a substantial number of c-FOS-labelled neurons (Figure 1C). Altogether, these results indicate that the effect of muscimol was mainly restricted to the DPGi.

**Figure 2:**
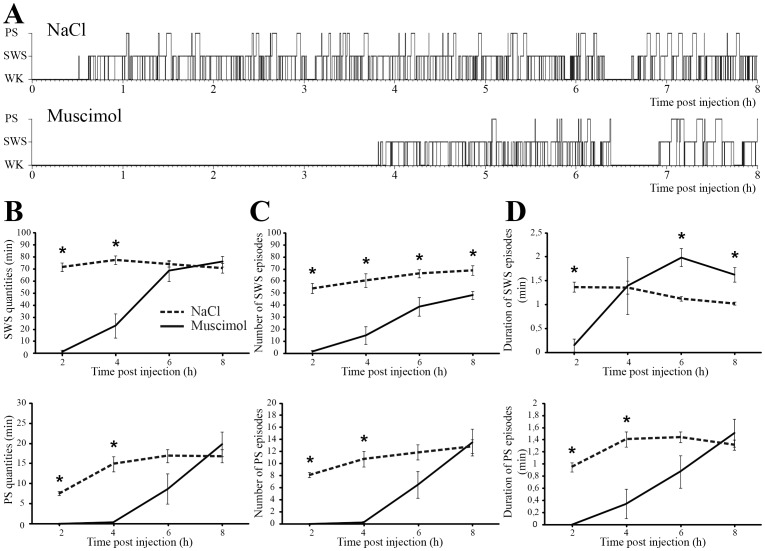
Muscimol inhibition of the DPGi induces waking and causes a prolonged suppression of PS. **A**, Hypnograms representing the sleep-wake cycle following control (NaCl) and muscimol injections in the DPGi in one representative animal. **B–D**, Effect of muscimol (black line) or NaCl (black dashed line) applications in the DPGi on PS and SWS quantities (**B**), number of PS and SWS episodes (**C**), duration of PS and SWS episodes (**D**). Values are mean ± SEM, n = 8 in each group, *p<0.05 between muscimol and NaCl.

During the first 4 h after muscimol injection, animals were mostly awake compared to NaCl (215.2±11.7 versus 68.4±5.4 min of waking, p = 0.01). During the next 4 h, WK quantities returned to control values (data not shown). SWS was nearly abolished during the first 2 h (1.5±1.5 min vs 71.7±3.7 min for NaCl, p = 0.01) and significantly reduced during the next 2 h (23±10.2 min vs 77.4±3.6 min for NaCl, p = 0.01) (Figure *2A–B*). The reduction in SWS quantities was due to a decrease in both the number and the duration of SWS episodes (Figure 2*C–D*). During the next 4 hours, SWS quantities returned to control values (p = 0.67, Figure 2*B*). PS was abolished during the first 4 h following muscimol injection (0.3±0.2 min vs 22.5±2 min for NaCl, p = 0.01) (Figure 2*B*). During the next 2 h block, PS quantities were reduced by muscimol treatment compared to NaCl but without reaching significance (8.7±3.8 min vs 16.9±1.6 min, p = 0.06). For the last 2 h of recordings, PS quantities returned to control values (19.9±3.1 min vs 15.3±1.3 min for NaCl, p = 0.3). SWS and PS latencies were increased after muscimol treatment as compared to NaCl, by 8.7 times (from 14.7±2.9 min to 177.2±27 min, p = 0.01) and 6 times (from 51.7±3.8 min to 299.1±25.1 min, p = 0.01), respectively. Importantly, the latency between the first SWS and the first PS episode was multiplied by 3 (from 37.1±4.6 min to 121.9±36.6 min, p = 0.01) after muscimol injection compared to NaCl.

**Figure 3:**
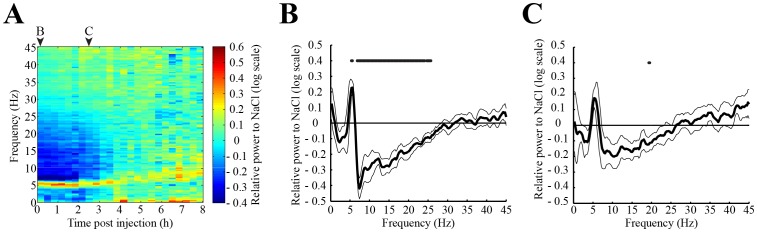
Spectral characterization of the sustained waking state induced by muscimol injection in the DPGi. **A**, Group data of time-frequency analysis of EEG signal during the 8 h following muscimol injection in the DPGi. The relative power in each frequency bin after muscimol treatment was normalized to NaCl values and represented using a logarithmic scale. The values of the two 20 min blocks highlighted by letters B and C in panel **A** are represented in **B** and **C**. Values are mean ± SEM, n = 7 in each group. The black points above the graphs show the frequency bins significantly different between the two conditions (p<0.05).

Waking induced by muscimol injection in the DPGi was characterized by a persistent and significant increase in the power of the theta band (5 – 6 Hz) and a decrease in the higher 7 – 25 Hz band (Figure 3*A,B*) when compared to WK after NaCl. These modifications lasted 140 min following muscimol injection (Figure 3*C*). During this period, visual examination of the animals showed that they are calm, almost immobile and apparently highly attentive to external stimuli.

### Clonidine Injection into the DPGi Selectively Reduces PS

**Figure 4:**
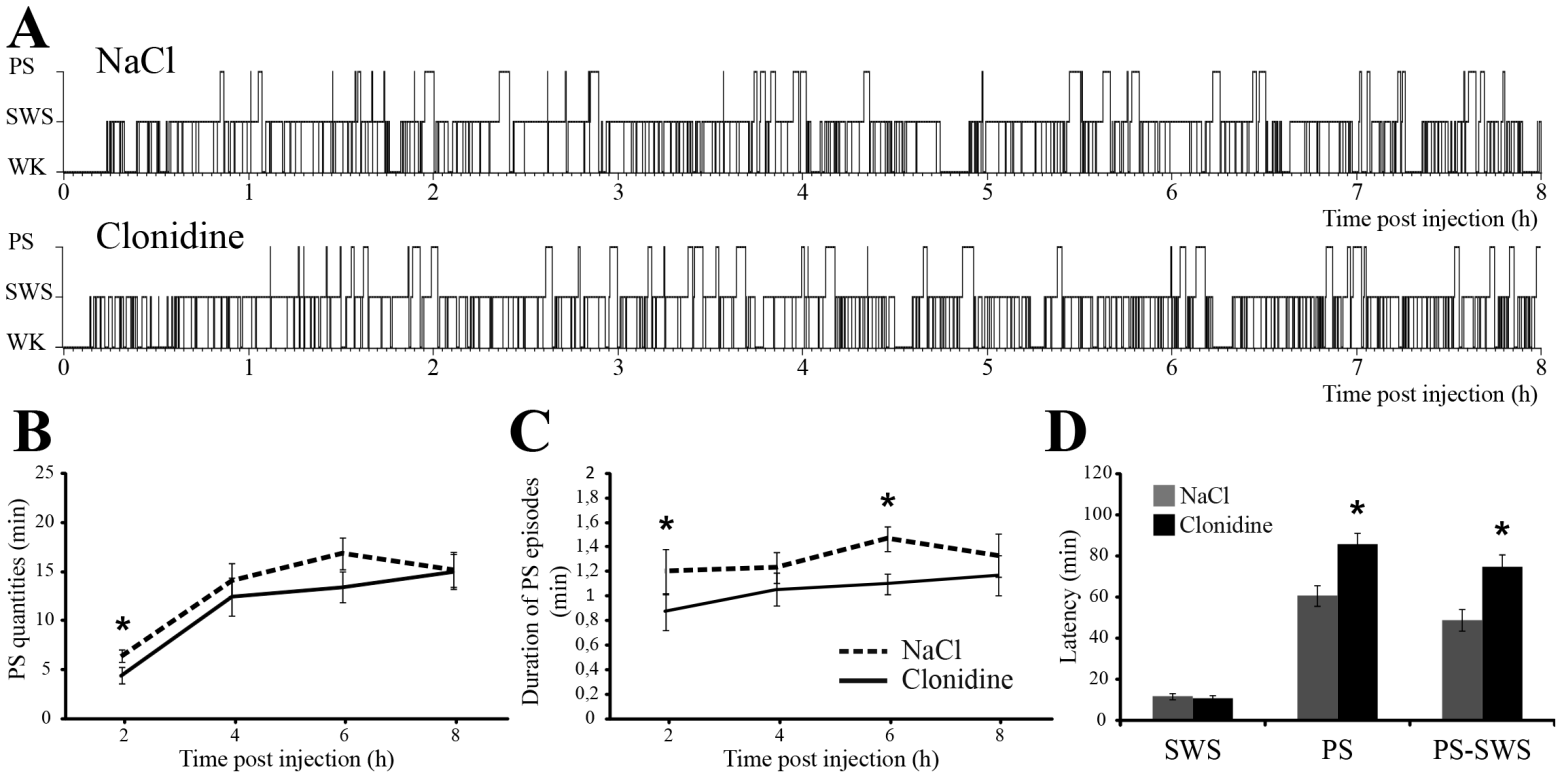
Clonidine injection in the DPGi specifically suppresses PS. **A**, Hypnograms representing the sleep-wake cycle following control (NaCl) and muscimol injections in the DPGi in one representative animal. **B–C**, Effect of clonidine (black line) or NaCl (black dashed line) applications in the DPGi on PS quantities (**B**) and duration of PS episodes (**C**). SWS and PS latencies, as well as the time between the first SWS and the first PS episode are shown in **D**. (n = 8 in each group). Values are mean ± SEM, *p<0.05 between muscimol and NaCl.

PS quantities were significantly reduced during the first 2 h after clonidine injection compared to control (4.4±0.8 min, vs 6.4±0.6 min, p = 0.049). A tendency to decrease was also observed during the 4 – 6 h block but it did not reach significance (13.4±1.6 min vs 16.9±1.7 min, p = 0.12) (Figure 4*B*). The duration (Figure 4*C*) but not the number (data not shown) of PS episodes was significantly decreased during the 0 – 2 h and the 4 – 6 h blocks. Moreover, the latency to the first PS but not to the first SWS episode was increased after clonidine injection compared to NaCl (from 60.6±5 min to 85.9±5.6 min, p = 0.01, Figure 4*D*). Further, the latency between the first SWS and the first PS episode was significantly increased after clonidine injection compared to NaCl (75.1±5.7 vs 48.8±5.2, p = 0.01). The effect of clonidine was specific to PS since the quantities of SWS and WK and the number and the mean duration of SWS and WK episodes were not different between clonidine and NaCl injections across the 8 h of recording (p>0.05 for all 2 h blocks, data not shown).

### c-FOS Staining of the Wake Promoting Neurons after Muscimol Injection into the DPGi

Since the expression of c-FOS is maximum 90 to 120 min after a stimulus [18], rats were perfused 3 h after a muscimol (n = 4) or a NaCl (n = 4) injection in the DPGi. The rats with a muscimol injection in the DPGi stayed mostly awake during the 3 h following muscimol injection (177.7±2.4 min). On the contrary, NaCl injected animals spent 45.7±4.9 min in WK, 112.1±3.2 min in SWS and 15.3±3.9 min in PS.

#### Noradrenergic and adrenergic neurons

**Figure 5:**
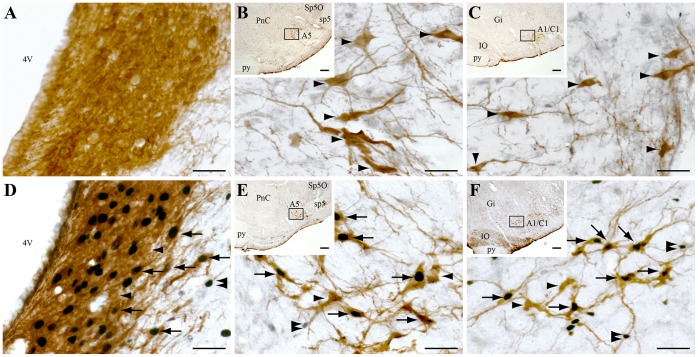
Noradrenergic and adrenergic neurons strongly express c-FOS after muscimol inhibition of the DPGi. Double-immunostained sections with TH (brown cytoplasmic staining) and c-FOS (black nuclear staining) at the level of A6 (**A**,**D**), A5 (**B**,**E**) and A1/C1 (**C**,**F**) noradrenergic cell groups after NaCl (top panel) or muscimol (bottom panel) injection in the DPGi. Black arrows point out double labeled neurons whereas black arrowheads and double arrowheads indicate TH singly and c-FOS singly labeled neurons, respectively. Scale bars, 50 µm for low power and 200 µm for high power photomicrographs in **B**, **C**, **E** and **F.** Abbreviations: 4V, 4th ventricle; A1/C1, A1 noradrenergic and C1 adrenergic groups; A5, A5 noradrenergic group; Gi, gigantocellular reticular nucleus; IO, inferior olive; PnC, pontine reticular nucleus, caudal part; py, pyramidal tract; sp5, spinal trigeminal tract; Sp5O, spinal trigeminal nucleus, oral part.

The number of pontine and medullary TH+ and TH/c-FOS+ neurons counted in animals with NaCl and muscimol injections are shown Table 1. In all but the C3 adrenergic group, the mean number of TH/c-FOS+ neurons is significantly and strongly increased after muscimol injections compared to NaCl (Table 1). In the LC (A6 noradrenergic group), 74% of the TH+ cells expresses c-FOS after muscimol treatment (Figure 5*A,D*). A similar activation of the other noradrenergic and adrenergic groups is observed in muscimol treated animals as 75% of A7, A2/C2 and A5 (Figure 5*B,E*), and 83% of A1/C1 (Figure 5*C,F*) TH+ cells express c-FOS after muscimol injection in the DPGi. In contrast, only 4% of the TH+ cells located in the C3 adrenergic group express c-FOS after muscimol injection (*Table 1*). The absence of activation of C3 adrenergic neurons after muscimol injection in the DPGi is not surprising since they are included in the injection site.

**Table 1:** Number and percentage of single and double-labeled neurons for c-FOS and the specific markers of the different waking systems after NaCl or muscimol injections in the DPGi.

		Mean number of all specific marker-labeled cells			Mean number of specific marker-labeled cells that were also c-FOS+			% of specific marker/c-FOS double labeled cells		
	n	NaCl	Muscimol	p	NaCl	Muscimol	p	NaCl	Muscimol	p
**Noradrenergic/Adrenergic (TH+)**										
A1/C1	5	103,5±12,9	117,8±8,4	0,248	13,3±4,7	93,5±6,6	0,021*	11,7±3,8	79,6±3,0	0,021*
A2/C2	4	58,0±6,7	47,3±8,2	0,312	8,3±2,4	34,0±4,3	0,021*	14,4±4,2	75,6±9,3	0,021*
A5	3	46,8±3,6	43,3±1,8	0,248	9,8±2,1	32,0±3,8	0,021*	21,3±4,5	75,1±10,5	0,021*
A6	3	202,0±11,9	244,0±25,9	0,194	21,0±9,6	174,8±20,8	0,021*	10,3±4,8	71,4±2,8	0,021*
A7	1	23,5±0,9	17,8±2,1	0,085	1,3±0,6	13,5±2,2	0,021*	5,1±2,5	75,0±5,1	0,021*
C3	2	18,5±3,2	21,8±1,3	0,248	1,3±0,6	1,8±0,9	0,665	5,9±2,2	7,9±4,1	0,885
**Dopaminergic (TH+)**										
A11	1	13,8±1,4	13,5±3,3	0,885	1,5±0,9	1,3±0,9	0,665	10,4±5,7	14,0±12,1	0,665
A9	1	236,8±13,0	190,3±19,1	0,773	7,0±1,1	7,0±2,7	0,564	3,0±0,6	3,6±1,2	0,387
A10	1	464,3±61,8	391,0±20,2	0,387	13,5±2,9	11,0±0,7	0,248	2,8±0,5	2,8±0,1	0,564
**Serotoninergic (5-HT+)**										
DR	4	308,8±31,7	228,8±27,0	0,149	7,0±2,5	40,3±14,2	0,043*	2,1±0,6	17,3±4,7	0,021*
MnR	4	137,3±7,0	112,5±17,6	0,564	3,5±1,2	16,0±4,1	0,021*	2,5±0,8	14,3±2,5	0,021*
B9	4	103,3±25,1	99,8±19,7	0,999	1,5±1,0	9,8±2,0	0,021*	1,6±1,0	11,5±3,4	0,043*
B3	3	32,0±3,8	28,5±6,2	0,387	0,8±0,5	16,0±8,6	0,030*	2,9±2,0	47,2±18,2	0,021*
RMg	3	53,8±1,8	56,3±3,5	0,470	3,8±2,4	12,5±3,8	0,083	7,2±4,8	22,1±6,3	0,083
ROb	4	101,8±1,9	112,3±11,6	0,999	4,0±2,1	1,0±0,4	0,470	3,9±2,1	0,9±0,3	0,470
RPa	4	43,8±6,5	40,0±7,2	0,564	6,8±2,3	6,0±2,1	0,773	15,1±5,0	17,0±5,6	0,773
**Cholinergic (ChAT+)**										
HDB	3	245,5±35,7	171,3±11,9	0,083	7,3±1,1	48,8±6,5	0,021*	3,1±0,6	28,5±3,5	0,021*
MCPO	3	358,3±40,2	333,0±21,1	0,387	24,0±1,3	138,5±17,4	0,021*	7,1±1,3	41,3±3,3	0,021*
MS	3	287,0±10,9	244,5±16,3	0,083	2,5±0,6	50,8±6,5	0,021*	0,9±0,2	20,7±2,3	0,021*
SI	6	284,3±17,1	310,0±26,4	0,387	2,3±1,0	51,5±6,1	0,021*	0,8±0,4	17,0±2,4	0,021*
PPTg	3	129,5±23,6	105,0±15,8	0,387	0,3±0,3	6,5±2,1	0,030*	0,2±0,2	6,5±2,3	0,021*
LDTg	3	325,0±22,4	287,3±22,9	0,248	2,0±0,6	38,3±9,6	0,021*	0,6±0,2	13,0±2,8	0,021*
**Hypocretinergic (HCRT+)**										
	3	299,0±12,7	262,5±18,8	0,112	57,8±3,7	145,5±22,3	0,021*	19,5±1,6	55,0±6,8	0,021*
**Histaminergic (HDC+)**										
TMN	2	90,8±15,1	109,3±8,9	0,248	15,5±10,6	55,0±6,0	0,043*	13,6±7,3	50,0±2,2	0,021*

Each value represents a mean ± SEM number of neurons per structure per side of the brain (n = 4 animals per group). The percentages displayed correspond to the number of double-labeled neurons versus the total number of neurons labeled with the marker of a given waking system. The number of sections counted for each structure is indicated in the column n. For each comparison the p value after Mann-Whitney *U* test is given in the column p. *p<0.05 between muscimol and NaCl.

Abbreviations: DR, dorsal raphe nucleus; HDB, nucleus of the horizontal limb of the diagonal band; LDTg, laterodorsal tegmental nucleus; MCPO, magnocellular preoptic nucleus; MnR, median raphe nucleus; MS, medial septal nucleus; PPTg, pedunculopontine tegmental nucleus; RMg, nucleus raphe magnus; ROb, nucleus raphe obscurus; RPa, nucleus raphe pallidus; TMN, tuberomammillary nucleus, ventral part.

#### Hypocretinergic and histaminergic neurons

**Figure 6:**
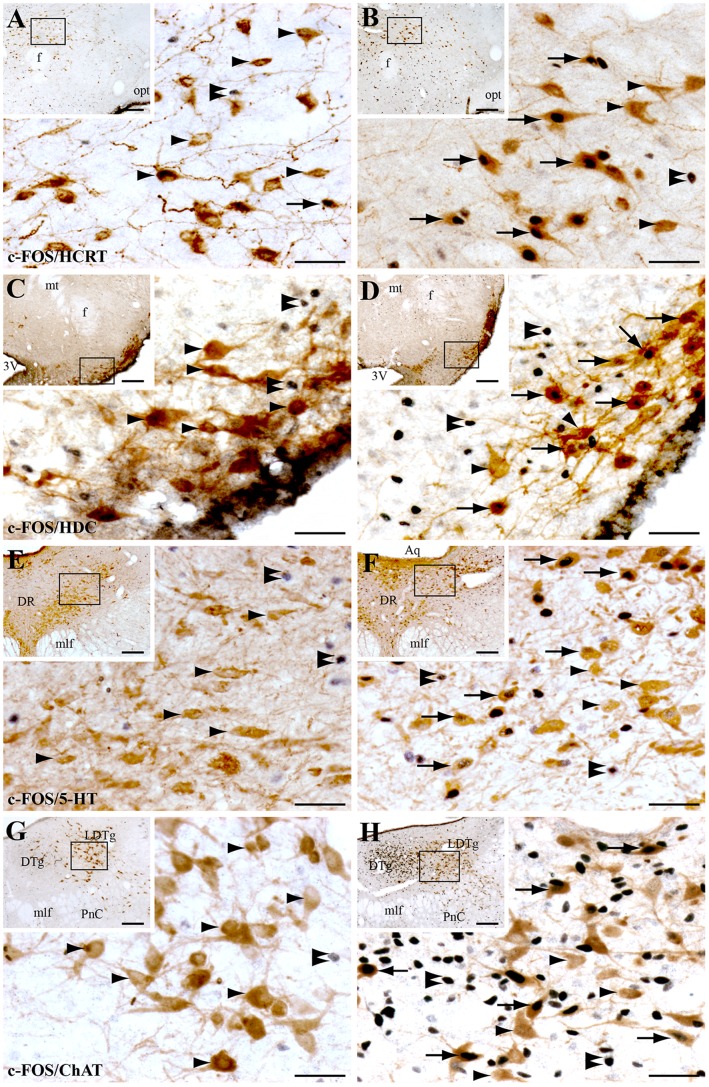
Level of c-FOS expression in wake promoting systems after NaCl or muscimol injection in the DPGi. Photomicrographs showing double immunostained sections for c-FOS (black nuclear staining) and hypocretin (HCRT, **A**, **B**), histamine (HDC, **C**, **D**), serotonin (5-HT, **E**, **F**) and choline acetyltransferase (ChAT, **G**, **H**) (brown cytoplasmic staining) 3 h after NaCl (left panel) or muscimol (right panel) injection in the DPGi. The areas illustrated at high power magnification are depicted in a box on the low power photomicrographs. Black arrows point out double-labeled neurons. Black arrowheads show singly labeled neurons for HCRT, HDC, 5-HT or ChAT and double arrowheads c-FOS singly labeled neurons. Scale bars, 50 µm for high enlargements and 200 µm for low enlargements**.** Abbreviations: 3V, 3rd ventricle; Aq, aqueduct; DR, dorsal raphe; DTg, dorsal tegmental nucleus; f, fornix; LDTg, laterodorsal tegmental nucleus; mlf, medial longitudinal fasciculus; mt, mammillothalamic tract; opt, optical tract; PnC, pontine reticular nucleus, caudal part.

As compared to control, muscimol injection in the DPGi induces a strong and significant activation of hypocretin and histaminergic neurons (Table 1). In muscimol treated animals, the percentage of double labeled neurons is of 61% for HCRT+ (Figure 6A,B) and 51% for histidine decarboxylase positive (HDC+) (Figure 6C,D) neurons.

#### Cholinergic neurons

The number of c-FOS and ChAT double-labeled neurons is significantly increased in the forebrain and to a lesser extent, but still significantly, in the brainstem after muscimol injection compared to NaCl (Table 1). Indeed, 44% of the ChAT+ neurons in the magnocellular preoptic nucleus and 30% of those of the medial septal nucleus, the horizontal limb of the diagonal band and the posterior part of the substantia innominata are c-FOS+ after muscimol injection. In contrast, only 9% and 11% of the ChAT+ neurons located in the pedunculopontine tegmental nucleus (PPTg) and in the laterodorsal tegmental nucleus (LDTg) express c-FOS, respectively (Figure 6*E,F*).

#### Serotoninergic neurons

The number of c-FOS and 5-HT double-labeled neurons is significantly increased in the dorsal and median raphe nuclei and the B3 and B9 serotonergic groups but not in the medullary raphe nuclei after muscimol injection compared to NaCl (Table 1). However, only a small percentage of 5-HT+ neurons were c-FOS+ after muscimol injections (Table 1). Indeed, the medullary serotoninergic group B3 was the only group in which a substantial proportion of 5-HT+ neurons were c-FOS+ (27%). In the other serotoninergic groups with a significant increase in the number of c-FOS/5-HT double-labeled cells, i.e. the B9 serotoninergic groups, the dorsal (DR) and median (MnR) raphe, less than 13% of the 5-HT+ neurons were double-labeled with c-FOS (Table 1 and Figure 6*G,H*).

#### Dopaminergic neurons

The number of double-labeled neurons in the mesencephalic and hypothalamic dopaminergic groups was not different between muscimol and NaCl animals. Indeed, in both experimental conditions, TH/c-FOS+ neurons represented less than 5% in A9 and A10, and less than 14% in A11 dopaminergic cell group of the TH+ neurons (Table 1).

#### Comparison of the percentage of double-labelled cells across groups

The percentage of double labelled cells in A1/C1, A2/C2, A5, A6 and A7 TH+ groups was significantly higher (p<0.05 for all comparison) than in HDC+, ChAT+, and 5-HT+ groups for all structures analyzed, excepting for the 5HT+ neurons in B3 group (p>0.05). The percentage of double labelled TH+ neurons was also significantly higher in A1/C1 (p = 0.021), A5 (p = 0.034), A7 (p = 0.043), but not in A6 (p = 0.083) and A2/C2 (p = 0.149) groups compared to HCRT+ neurons.

These statistical analyses indicate that muscimol inhibition of the DPGi induces a preferential increase in c-FOS expression in A1/C1, A2/C2, A5, A6 and A7 groups, followed by HCRT and HDC systems, and to a lesser extent 5-HT and ChAT neuronal groups.

## Discussion

### Methodological Consideration

It is well established that noradrenergic neurons of the central nervous system are very reactive to stressful stimuli (for review see [19], [20]). However, it is unlikely that the activation of noradrenergic and adrenergic neurons reported in this study is due to an acute stress. Indeed, animals were habituated during many days to the recording conditions, handling and injection procedure. Moreover, injections of the different pharmacological agents were randomized in order to prevent experimental bias and animals expressed normal behaviour during all injections, including that of muscimol.

It is also likely that the results reported here mainly result from the pharmacological inactivation of DPGi neurons. Indeed, as illustrated in Figure 1, all injection sites were localized in the middle of the DPGi and the volume injected was comparable to that used in previous studies [21]–[23]. Moreover, the analysis of c-FOS expression following muscimol injection revealed that, compared to NaCl, the number of c-FOS+ neurons was significantly and strongly reduced specifically in the DPGi. These results strongly suggest that muscimol diffusion was mainly restricted to the DPGi.

In order to assess the neuronal activation after pharmacological treatment, we chose to use the c-FOS method. Indeed, although c-FOS is not a perfect marker of neuronal activation as its expression does not strictly correlate with neuronal discharge [18], [24], it has been extensively used to map the neuronal circuitry controlling the sleep-wake cycle [11], [25]–[30] as well as the activation of TH+, 5-HT+, ChAT+, HCRT+ and HDC+ neurons following diverse experimental paradigms [31]–[41]. C-FOS labelling was not performed after clonidine injection since we believe that the decrease of PS obtained was not strong enough to induce c-FOS expression in the neurons inhibiting PS.

### Functional Significance

In this study, we report that the inactivation of the DPGi by muscimol induces a long lasting induction of waking. We also show that application of clonidine in the DPGi selectively inhibits PS. We further demonstrate using c-FOS that, during waking induced by muscimol injection in the DPGi, nearly all the noradrenergic and adrenergic neurons are activated in contrast to the other waking systems which show a significantly weaker activation. In the following, we discuss our results with regards to the literature before focusing on their physiological significance.

To the best of our knowledge, this study is the first to report that SWS and PS are totally suppressed following injection of muscimol in the DPGi. Importantly, muscimol inhibition of the DPGi more profoundly affects PS than SWS as the latency between the first SWS episode and the first PS episode was strongly increased following muscimol application as compared to control. Moreover, the application of clonidine inside the DPGi specifically decreases PS quantities without altering SWS. These results strongly suggest that DPGi neurons activity is required for sleep to occur. To find out the mechanisms involved, we examined the activity of the waking systems after muscimol injections. We found out that around 75% of the neurons of all noradrenergic and adrenergic groups, 55% of the hypocretin, 50% of the histaminergic, 17 to 41% of the basal forebrain cholinergic neurons and less than 20% of the serotoninergic, dopaminergic and pontine cholinergic neurons were c-FOS+ after muscimol injection. In contrast, after 2 to 6 h of waking induced by sleep deprivation or exposure to a new environment, only 30% of the noradrenergic and adrenergic neurons were c-FOS+, while 60% of the hypocretin neurons, 80% of the histaminergic neurons but less than 20% of the basal forebrain cholinergic neurons and 5% of the dopaminergic neurons expressed c-FOS [36], [37], [42]–[45]. To our knowledge, percentages of c-FOS labelled neurons have never been reported during sleep deprivation for the serotoninergic and pontine cholinergic neurons The fact that after DPGi inhibition, and not after waking induced by sleep deprivation or exposure to a new environment, nearly all adrenergic and noradrenergic neurons are c-FOS labelled while the percentage of c-FOS+ in the other waking neurons is lower suggest that the function of, the DPGi neurons might be to directly and specifically inhibit the activity of the noradrenergic and adrenergic neurons. A number of results support such hypothesis.

First, the strong inhibitory projection of the DPGi on the A6 noradrenergic neurons is well established. It has indeed been shown that the DPGi provides a strong projection to A6 [46] and that electrical stimulation of the DPGi induces with a short latency a GABAergic inhibition of A6 noradrenergic neurons [9]. It has also been shown that GABA release in the LC increases from waking to SWS and further during PS [7] and that application of bicuculline, a GABA_A_ antagonist on LC noradrenergic neurons increases their activity during waking but also restores a tonic firing when applied during SWS and PS [8]. Moreover, the DPGi contains a large number of GABAergic neurons activated during PS [6] and projecting to the LC [11], and unitary recordings in head-restrained rats showed that the DPGi contains neurons active during WK (4.24±0.9 Hz) progressively increasing their activity from SWS (7.34±1.1 Hz) to PS (46.06±5.1 Hz) [5]. These neurons increase their firing rate about 15 s before PS onset [5], whereas A6 noradrenergic WK-on neurons stop firing 10 – 20 s before PS onset [12], [47], [48]. Finally, electrical stimulation of the DPGi enhances PS [10]. From these and our results, it can be concluded that DPGi GABAergic neurons tonically inhibit LC noradrenergic neurons during PS but that they could also dampen their activity during SWS and WK. Our results also suggest that, in addition to A6, the DPGi could tonically inhibit the noradrenergic and adrenergic neurons of the other noradrenergic and adrenergic groups. Supporting this hypothesis, the DPGi strongly projects to the Kölliker-Fuse nucleus (containing A7 group), the lateral paragigantocellular nucleus (containing the A1/C1 group) and the nucleus of the solitary tract (containing the A2/C2 group) [5], [49], [50]. Furthermore, it is likely that all noradrenergic and adrenergic neurons share a common pattern of discharge across the sleep wake cycle, ie that they are active during WK, decrease their activity during SWS and are silent during PS. Indeed, it has been shown using electrophysiological recordings that neurons of the A5 noradrenergic neurons are totally silent during PS [51]. Further, the present c-FOS study and that of Leger et al [36] revealed that neurons of all noradrenergic and adrenergic groups are c-FOS positive during waking and c-FOS negative during PS hypersomnia.

A number of additional results strengthen our hypothesis that the inhibitory effect of the DPGi is rather specific to the adrenergic and noradrenergic neurons.

Indeed, although a majority of the hypocretin and the histaminergic neurons located in the hypothalamus are c-FOS+ after muscimol injection in the DPGi, the DPGi provides only a very small or no projection to these neurons [5]. Moreover, such percentages of c-FOS+ hypocretin and histaminergic neurons have been reported in rats sacrificed during the wake (dark) period [37], [45]. In view of these results, we propose that the activation of the hypocretin and histamine systems observed in this study is indirect and could result from their excitation by noradrenaline and/or adrenaline. Indeed, the hypocretin and the histaminergic neurons receive direct projections from noradrenergic and adrenergic cells [52], [53], and are excited by noradrenaline [54]–[56]. Finally, although the DPGi provides substantial projections to the dorsal raphe and the laterodorsal tegmental and pedunculopontine nuclei, serotoninergic and cholinergic neurons located in these structures are in their vast majority c-FOS negative after muscimol injections suggesting that they are not inhibited by the GABAergic DPGi neurons.

In the present study, we further demonstrate that clonidine injection in the DPGi specifically induces a decrease in PS quantities during the first two hours after injection and delays PS appearance compared to control. Since clonidine is a specific inhibitory alpha-2 adrenergic receptor agonist, these results first indicate that neurons of the DPGi bear alpha-2 adrenergic receptors. This is in agreement with previous studies showing the presence of alpha2 adrenergic receptors [14], [15] and that of DBH-immunoreactive fibers (dopamine β-hydroxylase, synthesising hormone) [57], [58] in the DPGi. Although it remains to be directly demonstrated, we therefore propose that GABAergic neurons of the DPGi express alpha-2 adrenergic receptors and are inhibited by noradrenaline. These results suggest that there is a reciprocal inhibitory interaction between the wake-active adrenergic and noradrenergic neurons and the GABAergic neurons of the DPGi. Such reciprocal inhibitory interaction could play a role in the swing between waking, slow wave and paradoxical sleep [3], [59].

### Conclusion: a New Integrated Model for the Genesis of the Sleep-waking Cycle

**Figure 7:**
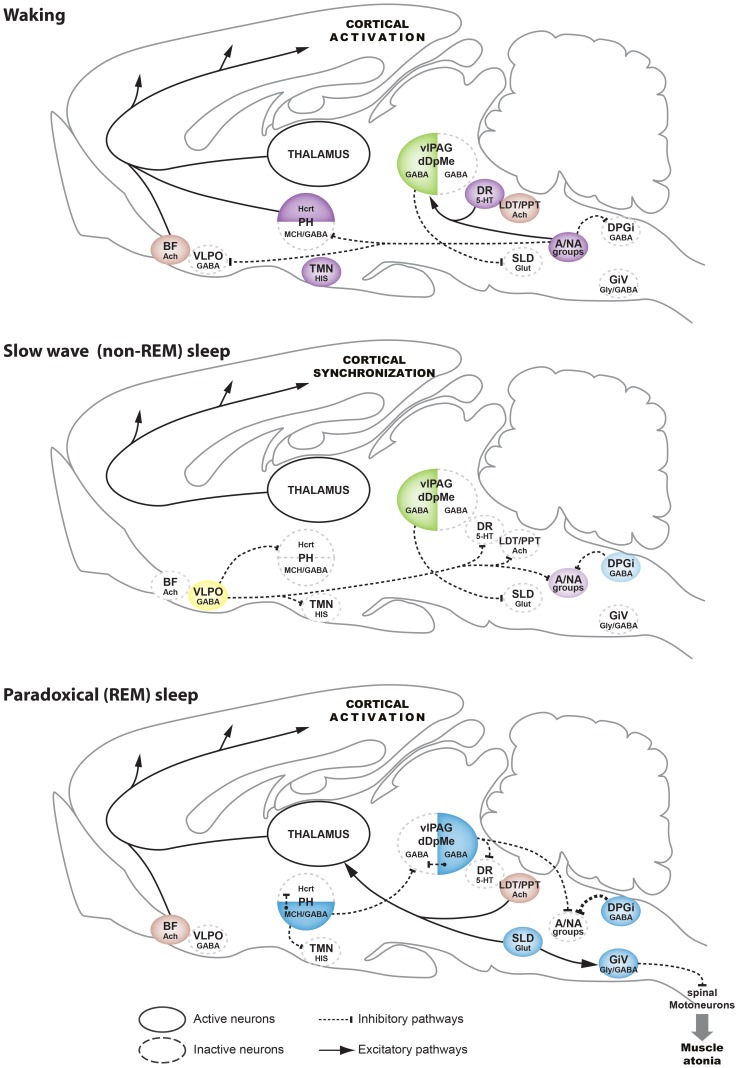
Illustration of the role of the DPGi in the network generating the sleep-waking cycle. During waking, the adrenergic/noradrenergic groups (A/NA) are active. They tonically inhibit the DPGi GABAergic and the posterior hypothalamic GABA/MCH PS-on neurons as well as the SWS-active GABAergic neurons of the VLPO (ventrolateral preoptic nucleus). They are also exciting the GABAergic PS-off neurons localized in the ventrolateral periaqueductal gray and the dorsal part of the deep mesencephalic nucleus (vlPAG/dDpMe) in charge of the inhibition of the sublaterodorsal tegmental (SLD) PS-on glutamatergic neurons. The other wake-active (hypocretin, histaminergic, serotoninergic) and W/PS (cholinergic) active systems are activated and excite the thalamocortical system. During SWS, the VLPO GABAergic neurons are activated and inhibit all wake-active neurons. The DPGi GABAergic neurons are slightly active and contribute to the inhibition of the A/NA neurons from all groups. The onset and maintenance of PS is due to the activation of three GABAergic PS-on neurons localized in the posterior hypothalamus, the vlPAG/dDpMe and the DPGi. Each of these systems is responsible for the inhibition of one or several populations of wake-active neurons and of the PS-off GABAergic neurons localized in the vlPAG/dDpMe. The role of the DPGi GABAergic neurons is to specifically inactivate during PS the A/NA neurons from all groups. The disinhibited SLD PS-on glutamatergic neurons generate cortical activation by their projections to the thalamus and muscle atonia by means of their descending projection to GABA/glycinergic neurons localized in the ventral gigantocellular reticular nucleus (GiV). These neurons hyperpolarize all somatic motoneurons.

This study reveals for the first time that the inactivation of the DPGi induces a long lasting period of WK. These results suggest that the activation of DPGi neurons might be necessary for sleep to occur. Based on our and previous results, we propose that GABAergic neurons of the DPGi provide an inhibitory drive to all noradrenergic and adrenergic neurons with an increasing strength from WK to SWS to PS. During SWS, they would contribute to the inhibition of the noradrenergic and adrenergic neurons in conjunction with the SWS-on GABAergic neurons localized in the ventrolateral preoptic area (VLPO) [3], [60] (Figure 7). DPGi GABAergic neurons would be responsible of the inhibition of noradrenergic and adrenergic neurons during PS with a minor contribution of the GABAergic neurons of the ventrolateral periaqueductal gray [2] (Figure 7). Reciprocally, our results showing that clonidine injection in the DPGi specifically inhibit PS suggest that the noradrenergic and adrenergic neurons inhibit DPGi GABAergic neurons by means of alpha-2 adrenergic receptors at the onset of and during waking. Interestingly, we recently showed that inhibition of the activity of the PS-on MCH/GABAergic neurons located in the posterior hypothalamus by application of clonidine also inhibits PS [61] and it is well know that SWS-generating neurons of the VLPO are inhibited by clonidine [62]. Altogether, these results suggest that adrenergic and noradrenergic neurons play a role in the onset of waking occurring after SWS and PS and its maintenance by means of their inhibitory projections to the VLPO SWS-active and the DPGi and posterior hypothalamic PS-active neurons, respectively (Figure 7).
